# Practical Notes on Diagnosis and Treatment

**Published:** 1907-11-30

**Authors:** 


### PRACTICAL NOTES ON DIAGNOSIS AND TREATMENT.

**Hiccough.**

Obstinate hiccough may sometimes be successfully checked by depression of the tongue by a spatula or spoon.

**Nocturnal Incontinence.**

A patient suffering from this condition may with advantage be made to take sleep for an hour or two in the middle of the day. The result is sometimes very good.

**Pulse Tension in Granular Kidney.**

In granular kidney it is better that the pulse tension should be high than low. Low tension is a bad sign; it occurs only in the later stage of the disease, though it may continue for some time.

**Idiopathic Ulcerative Colitis.**

Quinine in an initial dose of twenty grains and repeated in smaller doses every four hours is a useful drug in this condition. Salol with ʒj.—ʒij oil of turpentine in a pint of water is a useful enema.

**Pruritus Ani.**

Obstinate cases of pruritus ani sometimes depend on the presence of a small ulcer on the rectal mucous membrane between the external and internal sphincters. The best treatment is forcible stretching of the sphincter.

**Intra-urethral Chancre.**

The evidences of this may be very slight, often little more than a slight urethral discharge. Hence the condition may be regarded as a mild gonorrhœa, and its true nature may only become evident when symptoms of secondary syphilis appear.

**Turpentine in Hæmatemesis.**

Though in this condition neither food nor drugs should be given by the mouth, an exception may be made in favour of oil of turpentine. Of this ten minim doses emulsified with white of egg may be given and repeated, as may be necessary, every three hours or so.

**Tachycardia in Phthisis.**

A rapid pulse occurring apart from pyrexia is frequently one of the earliest signs of phthisis. Irritability of the pulse is another fact in early phthisis. Anything which naturally quickens the pulse does so to an exaggerated degree in commencing tubercle.

**Diabetic Coma.**

When coma threatens in diabetes transfusion of saline fluid should be promptly adopted; it should be used subcutaneously or directly injected into a vein. From three to four pints should be given in the course of twenty-four hours. A 0.7 per cent. solution of sodium chloride is suitable.

**Potassium Bichromate in Dyspepsia.**

Bichromate of potassium will often promptly remove such evidences of dyspepsia as anorexia, nausea, pain, vomiting, and gastric tenderness. It should be given on an empty stomach in doses of $\frac{1}{12}$ to $\frac{1}{6}$ grain three times a day. It may be ordered as a pill made with kaolin ointment, or in solution flavoured with syrup of tolu or orange. In gastric ulcer the results are just as good as in simple dyspepsia.

